# 2,2,3,3,5,5,6,6-Octa-*p*-tolyl-1,4-dioxa-2,3,5,6-tetra­germacyclo­hexane dichloro­methane disolvate

**DOI:** 10.1107/S1600536809032012

**Published:** 2009-08-19

**Authors:** Monika L. Amadoruge, Arnold L. Rheingold, Charles S. Weinert

**Affiliations:** a Department of Chemistry, Oklahoma State University, Stillwater, Oklahoma 74078, USA; b Department of Chemistry and Biochemistry, University of California San Diego, La Jolla, California 92092-0303, USA

## Abstract

The title compound, C_56_H_56_Ge_4_O_2_·2CH_2_Cl_2_ or Tol_8_Ge_4_O_2_·2CH_2_Cl_2_ (Tol = *p*-CH_3_C_6_H_4_), was obtained serendipitously during the attempted synthesis of a branched oligogermane from Tol_3_GeNMe_2_ and PhGeH_3_. The mol­ecule contains an inversion center in the middle of the Ge_4_O_2_ ring which is in a chair conformation. The Ge—Ge bond distance is 2.4418 (5) Å and the Ge—O bond distances are 1.790 (2) and 1.785 (2) Å. The torsion angles within the Ge_4_O_2_ ring are −56.7 (1) and 56.1 (1)° for the Ge—Ge—O—Ge angles and −43.9 (1)° for the O—Ge—Ge—O angle.

## Related literature

The related phenyl-substituted derivative Ph_8_Ge_4_O_2_ (Dräger & Häberle, 1985 ▶) is essentially isostructural with the title compound.
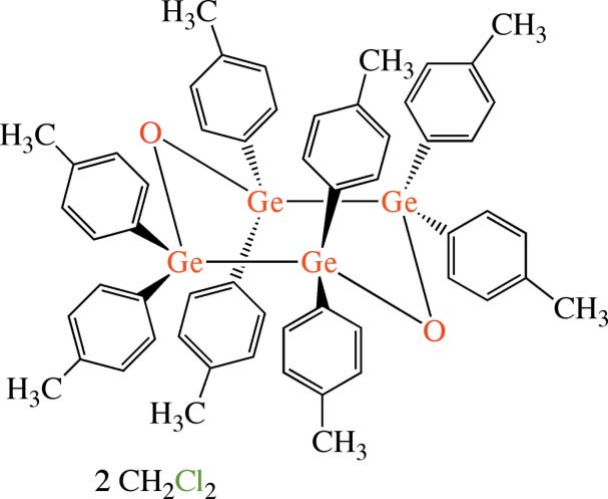


## Experimental

### 

#### Crystal data

C_56_H_56_Ge_4_O_2_·2CH_2_Cl_2_
*M* *_r_* = 1221.22
Triclinic, 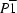

*a* = 10.781 (1) Å
*b* = 11.905 (1) Å
*c* = 12.295 (1) Å
α = 110.941 (1)°
β = 94.766 (1)°
γ = 109.069 (1)°
*V* = 1356.8 (2) Å^3^
*Z* = 1
Mo *K*α radiation
μ = 2.43 mm^−1^
*T* = 123 K
0.33 × 0.33 × 0.24 mm

#### Data collection

Bruker APEXII CCD diffractometer
Absorption correction: multi-scan (*SADABS*; Bruker, 2001 ▶) *T*_min_ = 0.471, *T*_max_ = 0.558
12912 measured reflections
5003 independent reflections
4484 reflections with *I* > 2σ(*I*)
*R*_int_ = 0.026

#### Refinement

*R*[*F* ^2^ > 2σ(*F* ^2^)] = 0.039
*wR*(*F* ^2^) = 0.128
*S* = 1.04
5003 reflections
307 parameters
H-atom parameters constrained
Δρ_max_ = 0.69 e Å^−3^
Δρ_min_ = −0.69 e Å^−3^

### 

Data collection: *APEX2* (Bruker, 2007 ▶); cell refinement: *SAINT* (Bruker, 2007 ▶); data reduction: *SAINT*; program(s) used to solve structure: *SHELXS97* (Sheldrick, 2008 ▶); program(s) used to refine structure: *SHELXL97* (Sheldrick, 2008 ▶); molecular graphics: *ORTEP-3 for Windows* (Farrugia 1997 ▶); software used to prepare material for publication: *SHELXTL* (Sheldrick, 2008 ▶).

## Supplementary Material

Crystal structure: contains datablocks I, global. DOI: 10.1107/S1600536809032012/lh2875sup1.cif

Structure factors: contains datablocks I. DOI: 10.1107/S1600536809032012/lh2875Isup2.hkl

Additional supplementary materials:  crystallographic information; 3D view; checkCIF report

## Figures and Tables

**Fig. 1.**
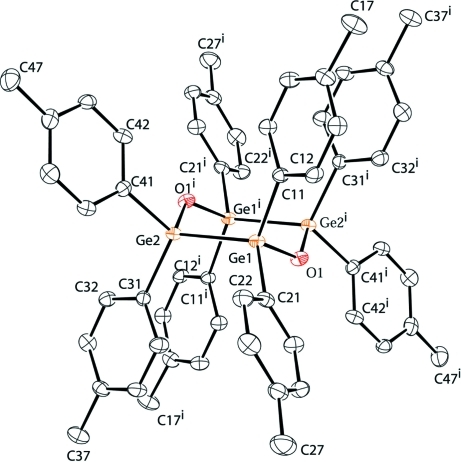
The molecular structure of (1), with displacement ellipsoids drawn at the 50% probability level. Primed atoms are related by the symmetry operator (-x, -y, -z+1).

**Fig. 2.**
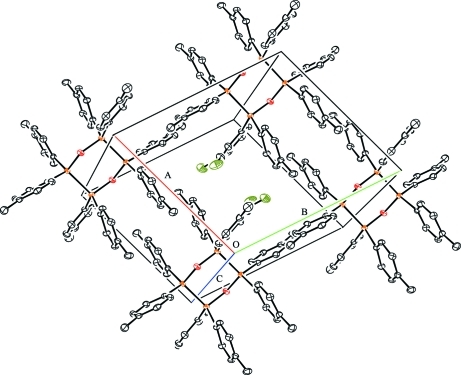
Part of the crystal structure of (1), viewed approximately along the c axis.

## supplementary crystallographic information

### Comment

The molecular structure of (1) is shown in Fig. 1. The molecule adopts approximate *C_2 h_* symmetry and has an inversion center located in the center of the Ge_4_O_2_ ring. The six- membered ring adopts a chair-like conformation analagous to that of its carbon-containing congener, 1,4-dioxane. The crystal structure of (1) can be compared to the essentially isostructural perphenyl-substituted derivative Ph_8_Ge_4_O_2_ (2) (Dräger *et al.*, 1985). The Ge-O distances of 1.790 (2) and  1.785 (2) Å in (1), are the same within experimental error as those in (2) (1.786 (1) and 1.781 (2) Å). The Ge - Ge single bond distance in (1) is 2.4418 (5) Å and is slightly shorter than that in (2) (2.448 (1) Å). The Ge1-C21 bond distance of 1.953 (3) Å is elongated relative to the remaining three Ge - C*_ipso_* bonds, which are all the same within experimental error. The Ge - C*_ipso_* bonds are nearly identical to those in the phenyl-substituted derivative (2).

The Ge1-O1-Ge2^i^ [symmetry code: (i) -x, -y, -z+1] angle of 126.7 (1) ° in (1) is the same, within experimental error, as that in (2) (126.9 (1) °), while the Ge1-Ge2-O1^i^ angle of 106.20 (8) ° is slightly smaller than that in (2) (106.7 (1)°). However, the Ge2-Ge1-O1 angle in (1) of 104.82 (8) °, is significantly smaller than that in (2) (106.7 (1) °). The torsion angles within the Ge_4_O_2_ ring in (1) are significantly different than those in (2). The Ge1-Ge2-O1^i^-Ge1^i^, Ge2^i^-O1-Ge1-Ge2, and O1-Ge1-Ge2-O1^i^ torsion angles are -56.7 (1), -56.1 (1), and 43.9 (1) ° (respectively), while the corresponding torsion angles in (2) are -53.1 (1), -53.1 (1), and 41.9 (1) °.

Part of the crystal structure is shown in Fig. 2. One germanium atom of two of the four symmetry related molecules shown lies within the selected unit cell, while a germanium atom and an oxygen atom in the remaining two molecules lie within this unit cell. The distances between the centroids of the Ge4O2 rings are 10.78 (1) Å parallel to the a axis and 111.91 (1) Å  parallel to the b axis.

### Experimental

The title compound (1) was unexpectedly obtained during the attempted preparation of (Tol_3_Ge)3GePh (Tol = *p*CH_3_C_6_H_4_) from Tol_3_GeNMe_2_ and PhGeH_3_. The crude reaction mixture was recrystallized from dichloromethane which yielded a three X-ray quality crystals, all of which were determined to be compound (1).

### Refinement

All hydrogen atoms were placed in calculated positions using a riding- model. Their positions were constrained realtive to their parent atom using the appropriate HFIX instruction in SHELXL97 (Sheldrick, 2008).

### Crystal data

**Table:**

C_56_H_56_Ge_4_O_2_·2CH_2_Cl_2_	*Z* = 1
*M**_r_* = 1221.22	*F*(000) = 620
Triclinic, *P*1	*D*_x_ = 1.495 Mg m^−^^3^
Hall symbol: -P 1	Mo *K*α radiation, λ = 0.71073 Å
*a* = 10.781 (1) Å	Cell parameters from 3385 reflections
*b* = 11.905 (1) Å	θ = 2.4–25.5°
*c* = 12.295 (1) Å	µ = 2.43 mm^−^^1^
α = 110.941 (1)°	*T* = 123 K
β = 94.766 (1)°	Block, colorless
γ = 109.069 (1)°	0.33 × 0.33 × 0.24 mm
*V* = 1356.8 (2) Å^3^	

### Data collection

**Table:**

Bruker APEXII CCD diffractometer	5003 independent reflections
Radiation source: fine-focus sealed tube	4484 reflections with *I* > 2σ(*I*)
graphite	*R*_int_ = 0.026
φ and ω scans	θ_max_ = 25.5°, θ_min_ = 1.8°
Absorption correction: multi-scan (*SADABS*; Bruker, 2001)	*h* = −12→13
*T*_min_ = 0.471, *T*_max_ = 0.558	*k* = −14→14
12912 measured reflections	*l* = −14→14

### Refinement

**Table:**

Refinement on *F*^2^	Primary atom site location: structure-invariant direct methods
Least-squares matrix: full	Secondary atom site location: difference Fourier map
*R*[*F*^2^ > 2σ(*F*^2^)] = 0.039	Hydrogen site location: inferred from neighbouring sites
*wR*(*F*^2^) = 0.128	H-atom parameters constrained
*S* = 1.04	*w* = 1/[σ^2^(*F*_o_^2^) + (0.085*P*)^2^ + 1.7*P*] where *P* = (*F*_o_^2^ + 2*F*_c_^2^)/3
5003 reflections	(Δ/σ)_max_ = 0.010
307 parameters	Δρ_max_ = 0.69 e Å^−^^3^
0 restraints	Δρ_min_ = −0.69 e Å^−^^3^

### Special details

**Table:**

Geometry. All e.s.d.'s (except the e.s.d. in the dihedral angle between two l.s. planes) are estimated using the full covariance matrix. The cell e.s.d.'s are taken into account individually in the estimation of e.s.d.'s in distances, angles and torsion angles; correlations between e.s.d.'s in cell parameters are only used when they are defined by crystal symmetry. An approximate (isotropic) treatment of cell e.s.d.'s is used for estimating e.s.d.'s involving l.s. planes.
Refinement. Refinement of *F*^2^ against ALL reflections. The weighted *R*-factor *wR* and goodness of fit *S* are based on *F*^2^, conventional *R*-factors *R* are based on *F*, with *F* set to zero for negative *F*^2^. The threshold expression of *F*^2^ > σ(*F*^2^) is used only for calculating *R*-factors(gt) *etc*. and is not relevant to the choice of reflections for refinement. *R*-factors based on *F*^2^ are statistically about twice as large as those based on *F*, and *R*- factors based on ALL data will be even larger.

### Fractional atomic coordinates and isotropic or equivalent isotropic displacement parameters (Å^2^)

**Table:**

	*x*	*y*	*z*	*U*_iso_*/*U*_eq_	
Ge1	−0.13908 (3)	−0.12172 (3)	0.54987 (3)	0.01478 (13)	
Ge2	0.05411 (3)	−0.15168 (3)	0.47168 (3)	0.01465 (13)	
Cl1	−0.42422 (12)	−0.66017 (13)	0.12064 (10)	0.0504 (3)	
Cl2	−0.34737 (13)	−0.62602 (13)	0.36550 (12)	0.0487 (3)	
O1	−0.0976 (2)	0.0503 (2)	0.6079 (2)	0.0176 (5)	
C11	−0.3071 (3)	−0.2118 (3)	0.4277 (3)	0.0159 (7)	
C12	−0.4291 (4)	−0.2575 (3)	0.4579 (3)	0.0186 (7)	
H12A	−0.4300	−0.2533	0.5365	0.022*	
C13	−0.5499 (4)	−0.3096 (3)	0.3745 (3)	0.0202 (7)	
H13A	−0.6325	−0.3394	0.3973	0.024*	
C14	−0.5521 (4)	−0.3189 (3)	0.2584 (3)	0.0207 (7)	
C15	−0.4299 (4)	−0.2780 (3)	0.2270 (3)	0.0216 (8)	
H15A	−0.4297	−0.2860	0.1473	0.026*	
C16	−0.3080 (4)	−0.2255 (3)	0.3089 (3)	0.0179 (7)	
H16A	−0.2255	−0.1990	0.2850	0.022*	
C17	−0.6840 (4)	−0.3656 (4)	0.1715 (4)	0.0301 (9)	
H17A	−0.6664	−0.3662	0.0944	0.045*	
H17B	−0.7309	−0.3071	0.2029	0.045*	
H17C	−0.7403	−0.4539	0.1606	0.045*	
C21	−0.1694 (3)	−0.1603 (3)	0.6886 (3)	0.0171 (7)	
C22	−0.2246 (4)	−0.2883 (4)	0.6779 (3)	0.0227 (8)	
H22A	−0.2491	−0.3583	0.6014	0.027*	
C23	−0.2442 (4)	−0.3144 (4)	0.7784 (3)	0.0259 (8)	
H23A	−0.2830	−0.4021	0.7695	0.031*	
C24	−0.2076 (4)	−0.2135 (4)	0.8919 (3)	0.0240 (8)	
C25	−0.1539 (4)	−0.0865 (4)	0.9010 (3)	0.0238 (8)	
H25A	−0.1294	−0.0162	0.9773	0.029*	
C26	−0.1353 (4)	−0.0601 (4)	0.8020 (3)	0.0214 (7)	
H26A	−0.0987	0.0278	0.8110	0.026*	
C27	−0.2218 (5)	−0.2411 (5)	1.0016 (4)	0.0352 (10)	
H27A	−0.1859	−0.1589	1.0729	0.053*	
H27B	−0.1715	−0.2951	1.0064	0.053*	
H27C	−0.3171	−0.2873	0.9967	0.053*	
C31	0.2103 (3)	−0.1027 (3)	0.5950 (3)	0.0161 (7)	
C32	0.3365 (4)	−0.0790 (3)	0.5677 (3)	0.0204 (7)	
H32A	0.3441	−0.0910	0.4882	0.024*	
C33	0.4503 (4)	−0.0386 (4)	0.6537 (3)	0.0215 (7)	
H33A	0.5353	−0.0224	0.6327	0.026*	
C34	0.4436 (4)	−0.0209 (3)	0.7708 (3)	0.0203 (7)	
C35	0.3173 (4)	−0.0456 (4)	0.7987 (3)	0.0220 (8)	
H35A	0.3103	−0.0356	0.8778	0.026*	
C36	0.2015 (4)	−0.0846 (4)	0.7134 (3)	0.0215 (8)	
H36A	0.1168	−0.0990	0.7348	0.026*	
C37	0.5681 (4)	0.0202 (4)	0.8630 (4)	0.0298 (9)	
H37A	0.5444	0.0278	0.9398	0.045*	
H37B	0.6327	0.1046	0.8715	0.045*	
H37C	0.6087	−0.0449	0.8379	0.045*	
C41	0.0141 (3)	−0.3236 (3)	0.3480 (3)	0.0168 (7)	
C42	−0.0299 (4)	−0.3508 (3)	0.2284 (3)	0.0192 (7)	
H42A	−0.0363	−0.2836	0.2066	0.023*	
C43	−0.0646 (4)	−0.4758 (4)	0.1403 (3)	0.0207 (7)	
H43A	−0.0940	−0.4926	0.0590	0.025*	
C44	−0.0568 (3)	−0.5761 (3)	0.1697 (3)	0.0223 (8)	
C45	−0.0114 (4)	−0.5482 (4)	0.2895 (3)	0.0234 (8)	
H45A	−0.0049	−0.6155	0.3111	0.028*	
C46	0.0242 (4)	−0.4241 (3)	0.3776 (3)	0.0196 (7)	
H46A	0.0557	−0.4070	0.4586	0.023*	
C47	−0.0987 (4)	−0.7119 (4)	0.0750 (4)	0.0312 (9)	
H47A	−0.1281	−0.7134	−0.0032	0.047*	
H47B	−0.1731	−0.7716	0.0926	0.047*	
H47C	−0.0222	−0.7393	0.0741	0.047*	
C51	−0.3605 (4)	−0.5477 (4)	0.2705 (4)	0.0302 (9)	
H51A	−0.2705	−0.4833	0.2789	0.036*	
H51B	−0.4207	−0.5003	0.2944	0.036*	

### Atomic displacement parameters (Å^2^)

**Table:**

	*U*^11^	*U*^22^	*U*^33^	*U*^12^	*U*^13^	*U*^23^
Ge1	0.0139 (2)	0.0158 (2)	0.0129 (2)	0.00301 (15)	0.00181 (15)	0.00655 (15)
Ge2	0.0143 (2)	0.0151 (2)	0.0125 (2)	0.00354 (16)	0.00122 (15)	0.00574 (15)
Cl1	0.0304 (6)	0.0638 (8)	0.0307 (6)	0.0060 (6)	0.0030 (5)	0.0020 (5)
Cl2	0.0510 (7)	0.0538 (7)	0.0555 (7)	0.0164 (6)	0.0221 (6)	0.0391 (6)
O1	0.0207 (13)	0.0147 (12)	0.0147 (12)	0.0039 (10)	0.0037 (10)	0.0058 (9)
C11	0.0143 (17)	0.0165 (16)	0.0146 (16)	0.0039 (13)	0.0026 (13)	0.0058 (13)
C12	0.0185 (18)	0.0235 (18)	0.0146 (16)	0.0067 (15)	0.0077 (14)	0.0089 (14)
C13	0.0158 (17)	0.0209 (18)	0.0232 (18)	0.0033 (14)	0.0070 (14)	0.0111 (15)
C14	0.0187 (18)	0.0153 (17)	0.0240 (18)	0.0022 (14)	−0.0007 (15)	0.0085 (14)
C15	0.0235 (19)	0.0221 (18)	0.0168 (17)	0.0052 (15)	0.0025 (15)	0.0090 (15)
C16	0.0161 (17)	0.0196 (17)	0.0170 (17)	0.0037 (14)	0.0051 (14)	0.0090 (14)
C17	0.018 (2)	0.036 (2)	0.033 (2)	0.0020 (17)	−0.0019 (17)	0.0200 (18)
C21	0.0119 (16)	0.0227 (18)	0.0175 (17)	0.0044 (14)	0.0029 (13)	0.0112 (14)
C22	0.026 (2)	0.0223 (18)	0.0194 (18)	0.0079 (16)	0.0033 (15)	0.0092 (15)
C23	0.027 (2)	0.025 (2)	0.031 (2)	0.0073 (16)	0.0075 (17)	0.0185 (17)
C24	0.0197 (19)	0.036 (2)	0.0212 (18)	0.0120 (17)	0.0053 (15)	0.0158 (17)
C25	0.023 (2)	0.030 (2)	0.0165 (17)	0.0112 (16)	0.0039 (15)	0.0070 (15)
C26	0.0202 (19)	0.0221 (18)	0.0193 (18)	0.0043 (15)	0.0043 (14)	0.0091 (15)
C27	0.035 (2)	0.052 (3)	0.028 (2)	0.018 (2)	0.0097 (18)	0.025 (2)
C31	0.0153 (17)	0.0155 (16)	0.0166 (17)	0.0063 (14)	0.0034 (13)	0.0053 (13)
C32	0.0214 (19)	0.0222 (18)	0.0169 (17)	0.0076 (15)	0.0050 (14)	0.0079 (14)
C33	0.0175 (18)	0.0218 (18)	0.0262 (19)	0.0053 (15)	0.0059 (15)	0.0127 (15)
C34	0.0183 (18)	0.0168 (17)	0.0217 (18)	0.0039 (14)	−0.0023 (14)	0.0073 (14)
C35	0.0220 (19)	0.0275 (19)	0.0163 (17)	0.0077 (16)	0.0022 (14)	0.0109 (15)
C36	0.0194 (19)	0.0264 (19)	0.0185 (18)	0.0058 (15)	0.0054 (15)	0.0111 (15)
C37	0.021 (2)	0.035 (2)	0.028 (2)	0.0066 (17)	−0.0039 (16)	0.0133 (18)
C41	0.0137 (17)	0.0174 (17)	0.0168 (17)	0.0045 (14)	0.0044 (13)	0.0053 (14)
C42	0.0178 (18)	0.0204 (17)	0.0186 (17)	0.0042 (14)	0.0004 (14)	0.0107 (14)
C43	0.0148 (17)	0.0275 (19)	0.0150 (17)	0.0065 (15)	0.0020 (14)	0.0052 (14)
C44	0.0112 (17)	0.0187 (18)	0.029 (2)	0.0027 (14)	0.0038 (15)	0.0036 (15)
C45	0.024 (2)	0.0192 (18)	0.028 (2)	0.0064 (15)	0.0054 (16)	0.0123 (16)
C46	0.0174 (18)	0.0219 (18)	0.0175 (17)	0.0054 (15)	0.0012 (14)	0.0085 (14)
C47	0.025 (2)	0.022 (2)	0.035 (2)	0.0076 (17)	0.0021 (17)	0.0014 (17)
C51	0.034 (2)	0.022 (2)	0.029 (2)	0.0070 (17)	0.0054 (18)	0.0082 (16)

### Geometric parameters (Å, °)

**Table:**

Ge1—O1	1.790 (2)	C26—H26A	0.950
Ge1—C21	1.945 (3)	C27—H27A	0.979
Ge1—C11	1.953 (3)	C27—H27B	0.980
Ge1—Ge2	2.4418 (5)	C27—H27C	0.979
Ge2—O1^i^	1.785 (2)	C32—H32A	0.951
Ge2—C41	1.944 (3)	C33—H33A	0.950
Ge2—C31	1.943 (3)	C35—H35A	0.949
Cl1—C51	1.756 (4)	C36—H36A	0.950
Cl2—C51	1.758 (4)	C37—H37A	0.979
O1—Ge2^i^	1.785 (2)	C37—H37B	0.980
C11—C12	1.386 (5)	C37—H37C	0.980
C11—C16	1.410 (5)	C42—H42A	0.950
C12—C13	1.389 (5)	C43—H43A	0.950
C13—C14	1.389 (5)	C45—H45A	0.950
C14—C15	1.387 (5)	C46—H46A	0.950
C14—C17	1.510 (5)	C47—H47A	0.979
C15—C16	1.388 (5)	C47—H47B	0.981
C21—C26	1.393 (5)	C47—H47C	0.981
C21—C22	1.394 (5)	C12—H12A	0.951
C22—C23	1.394 (5)	C13—H13A	0.951
C23—C24	1.395 (6)	C15—H15A	0.950
C24—C25	1.390 (5)	C16—H16A	0.950
C24—C27	1.505 (5)	C17—H17A	0.980
C25—C26	1.376 (5)	C17—H17B	0.980
C31—C32	1.391 (5)	C17—H17C	0.980
C31—C36	1.410 (5)	C22—H22A	0.951
C32—C33	1.375 (5)	C23—H23A	0.950
C33—C34	1.390 (5)	C25—H25A	0.950
C34—C35	1.395 (5)	C26—H26A	0.950
C34—C37	1.500 (5)	C27—H27A	0.979
C35—C36	1.388 (5)	C27—H27B	0.980
C41—C42	1.391 (5)	C27—H27C	0.979
C41—C46	1.402 (5)	C32—H32A	0.951
C42—C43	1.394 (5)	C33—H33A	0.950
C43—C44	1.391 (5)	C35—H35A	0.949
C44—C45	1.395 (5)	C36—H36A	0.950
C44—C47	1.505 (5)	C37—H37A	0.979
C45—C46	1.386 (5)	C37—H37B	0.980
C12—H12A	0.951	C37—H37C	0.980
C13—H13A	0.951	C42—H42A	0.950
C15—H15A	0.950	C43—H43A	0.950
C16—H16A	0.950	C45—H45A	0.950
C17—H17A	0.980	C46—H46A	0.950
C17—H17B	0.980	C47—H47A	0.979
C17—H17C	0.980	C47—H47B	0.981
C22—H22A	0.951	C47—H47C	0.981
C23—H23A	0.950	C51—H51A	0.990
C25—H25A	0.950	C51—H51B	0.989
			
O1—Ge1—C21	102.6 (1)	H33A—C33—C34	119.3
O1—Ge1—C11	109.6 (1)	C34—C35—H35A	119.2
C21—Ge1—C11	109.1 (1)	H35A—C35—C36	119.2
O1—Ge1—Ge2	104.82 (8)	C31—C36—H36A	120.2
C21—Ge1—Ge2	116.8 (1)	C35—C36—H36A	120.1
C11—Ge1—Ge2	113.1 (1)	C34—C37—H37A	109.5
O1^i^—Ge2—C41	102.3 (1)	C34—C37—H37B	109.5
O1^i^—Ge2—C31	108.8 (1)	C34—C37—H37C	109.5
C41—Ge2—C31	110.5 (1)	H37A—C37—H37B	109.5
O1^i^—Ge2—Ge1	106.20 (8)	H37A—C37—H37C	109.5
C41—Ge2—Ge1	114.5 (1)	H37B—C37—H37C	109.4
C31—Ge2—Ge1	113.7 (1)	C41—C42—H42A	119.7
Ge2^i^—O1—Ge1	126.7 (1)	H42A—C42—C43	119.8
C12—C11—C16	118.6 (3)	C42—C43—H43A	119.5
C12—C11—Ge1	120.3 (2)	H43A—C43—C44	119.6
C16—C11—Ge1	121.0 (3)	C44—C45—H45A	119.4
C13—C12—C11	120.7 (3)	H45A—C45—C46	119.4
C12—C13—C14	121.1 (3)	C41—C46—H46A	119.8
C13—C14—C15	118.1 (3)	C45—C46—H46A	119.8
C13—C14—C17	120.6 (3)	C44—C47—H47A	109.5
C15—C14—C17	121.2 (3)	C44—C47—H47B	109.4
C16—C15—C14	121.7 (3)	C44—C47—H47C	109.5
C15—C16—C11	119.6 (3)	H47A—C47—H47B	109.5
C26—C21—C22	118.2 (3)	H47A—C47—H47C	109.5
C26—C21—Ge1	120.6 (3)	H47B—C47—H47C	109.4
C22—C21—Ge1	121.2 (3)	C11—C12—H12A	119.6
C21—C22—C23	120.5 (3)	H12A—C12—C13	119.7
C22—C23—C24	120.9 (4)	C12—C13—H13A	119.4
C25—C24—C23	117.8 (3)	H13A—C13—C14	119.5
C25—C24—C27	121.0 (4)	C14—C15—H15A	119.1
C23—C24—C27	121.2 (4)	H15A—C15—C16	119.2
C26—C25—C24	121.5 (3)	C11—C16—H16A	120.1
C25—C26—C21	121.0 (3)	C15—C16—H16A	120.2
C32—C31—C36	118.4 (3)	C14—C17—H17A	109.4
C32—C31—Ge2	119.4 (2)	C14—C17—H17B	109.5
C36—C31—Ge2	122.2 (3)	C14—C17—H17C	109.5
C33—C32—C31	121.1 (3)	H17A—C17—H17B	109.5
C32—C33—C34	121.3 (3)	H17A—C17—H17C	109.4
C33—C34—C35	117.9 (3)	H17B—C17—H17C	109.5
C33—C34—C37	120.8 (3)	C21—C22—H22A	119.7
C35—C34—C37	121.3 (3)	H22A—C22—C23	119.8
C36—C35—C34	121.6 (3)	C22—C23—H23A	119.5
C35—C36—C31	119.7 (3)	H23A—C23—C24	119.5
C42—C41—C46	118.6 (3)	C24—C25—H25A	119.3
C42—C41—Ge2	120.6 (3)	H25A—C25—C26	119.2
C46—C41—Ge2	120.8 (2)	C21—C26—H26A	119.4
C41—C42—C43	120.6 (3)	C25—C26—H26A	119.6
C44—C43—C42	120.9 (3)	C24—C27—H27A	109.4
C43—C44—C45	118.3 (3)	C24—C27—H27B	109.4
C43—C44—C47	120.9 (3)	C24—C27—H27C	109.5
C45—C44—C47	120.7 (3)	H27A—C27—H27B	109.5
C46—C45—C44	121.1 (3)	H27A—C27—H27C	109.5
C45—C46—C41	120.5 (3)	H27B—C27—H27C	109.5
Cl2—C51—Cl1	111.2 (2)	C31—C32—H32A	119.4
C11—C12—H12A	119.6	H32A—C32—C33	119.5
H12A—C12—C13	119.7	C32—C33—H33A	119.3
C12—C13—H13A	119.4	H33A—C33—C34	119.3
H13A—C13—C14	119.5	C34—C35—H35A	119.2
C14—C15—H15A	119.1	H35A—C35—C36	119.2
C14—C15—C16	121.7	C31—C36—H36A	120.2
H15A—C15—C16	119.2	C35—C36—H36A	120.1
C11—C16—H16A	120.1	C34—C37—H37A	109.5
C15—C16—H16A	120.2	C34—C37—H37B	109.5
C14—C17—H17A	109.4	C34—C37—H37C	109.5
C14—C17—H17B	109.5	H37A—C37—H37B	109.5
C14—C17—H17C	109.5	H37A—C37—H37C	109.5
H17A—C17—H17B	109.5	H37B—C37—H37C	109.4
H17A—C17—H17C	109.4	C41—C42—H42A	119.7
H17B—C17—H17C	109.5	H42A—C42—C43	119.8
C21—C22—H22A	119.7	C42—C43—H43A	119.5
H22A—C22—C23	119.8	H43A—C43—C44	119.6
C22—C23—H23A	119.5	C44—C45—H45A	119.4
H23A—C23—C24	119.5	H45A—C45—C46	119.4
C24—C25—H25A	119.3	C41—C46—H46A	119.8
H25A—C25—C26	119.2	C45—C46—H46A	119.8
C21—C26—H26A	119.4	C44—C47—H47A	109.5
C25—C26—H26A	119.6	C44—C47—H47B	109.4
C24—C27—H27A	109.4	C44—C47—H47C	109.5
C24—C27—H27B	109.4	H47A—C47—H47B	109.5
C24—C27—H27C	109.5	H47A—C47—H47C	109.5
H27A—C27—H27B	109.5	H47B—C47—H47C	109.4
H27A—C27—H27C	109.5	Cl1—C51—H51A	109.4
H27B—C27—H27C	109.5	Cl1—C51—H51B	109.5
C31—C32—H32A	119.4	Cl2—C51—H51A	109.4
H32A—C32—C33	119.5	Cl2—C51—H51B	109.4
C32—C33—H33A	119.3	H51A—C51—H51B	108.0

Symmetry codes: (i) −*x*, −*y*, −*z*+1.
